# Supplementary material for: MetaQTL: a package of new computational methods for the meta-analysis of QTL mapping experiments
Source: BMC Bioinformatics. 2007 Feb 8;8:49. doi: 10.1186/1471-2105-8-49 (PMC1808479; doi:10.1186/1471-2105-8-49)
Supplement: Additional File 7 — MetaQTL Package : jar file and tutorial. This Zip archive contains both the MetaQTL JAR file and the files of the tutorial. [file 1471-2105-8-49-S7.zip › org.inra.metaqtl/doc/org/metaqtl/IMetaAlgorithm.html]

IMetaAlgorithm


|  |  |  |
| --- | --- | --- |
| **Overview** \| **Package** \| **Class** \| **Use** \| **Tree** \| **Deprecated** \| **Index** \| **Help** | |  |
| **PREV CLASS**   **NEXT CLASS** | **FRAMES**    **NO FRAMES**     **All Classes** |  |
| SUMMARY: NESTED \| FIELD \| CONSTR \| METHOD | DETAIL: FIELD \| CONSTR \| METHOD |  |


---


## org.metaqtl Interface IMetaAlgorithm

**All Known Implementing Classes:**: ClustAlgorithm, MetaAlgorithm, MetaMapAlgorithm, QtlClustAlgorithm, QtlProjAlgorithm, QtlTreeAlgorithm

---

``` public interface IMetaAlgorithm ```

This interface defines some methods which are common
to data analysis algorithms.

---

| **Method Summary** | |
| --- | --- |
| `int` | `getWorkAmount()`             Returns the amount of the work to do. |
| `int` | `getWorkProgress()`             Returns the amount of work which have already been done. |
| `void` | `run()`             Run the algorithm. |
| `void` | `setLogger(java.io.OutputStream stream)`             Give a `OutputStream` to the algorithm in order to write out the log info. |
| `void` | `setLogger(java.io.Writer writer)`             Give a `Writer` to the algorithm in order to write out the log info. |

**Method Detail**

### run

```
void run()
```

:   Run the algorithm.

---


### getWorkAmount

```
int getWorkAmount()
```

:   Returns the amount of the work to do.

    :   **Returns:**

---


### getWorkProgress

```
int getWorkProgress()
```

:   Returns the amount of work which have already
    been done.

    :   **Returns:**

---


### setLogger

```
void setLogger(java.io.Writer writer)
```

:   Give a `Writer` to the algorithm
    in order to write out the log info. The user
    is responsible for closing the stream.

    :   **Parameters:**: `writer` -

---


### setLogger

```
void setLogger(java.io.OutputStream stream)
```

:   Give a `OutputStream` to the algorithm
    in order to write out the log info. The user
    is responsible for closing the stream.

    :   **Parameters:**: `stream` -


---


|  |  |  |
| --- | --- | --- |
| **Overview** \| **Package** \| **Class** \| **Use** \| **Tree** \| **Deprecated** \| **Index** \| **Help** | |  |
| **PREV CLASS**   **NEXT CLASS** | **FRAMES**    **NO FRAMES**     **All Classes** |  |
| SUMMARY: NESTED \| FIELD \| CONSTR \| METHOD | DETAIL: FIELD \| CONSTR \| METHOD |  |


---
